# Pelvic Malignant Peripheral Nerve Sheath Tumor Revealing Previously Undiagnosed Familial Neurofibromatosis Type 1 in a Nine-Year-Old Girl: A Case Report

**DOI:** 10.7759/cureus.105592

**Published:** 2026-03-21

**Authors:** Hind Zahiri, Ayad Ghanam, Madiha Benhachem, Manal Azizi, Maria Rkain

**Affiliations:** 1 Department of Pediatric Medicine, Mohammed VI University Hospital, Faculty of Medicine and Pharmacy, Mohammed I University, Oujda, MAR; 2 Department of Pediatric Gastroenterology, Mohammed VI University Hospital Center, Oujda, MAR

**Keywords:** café au lait macules, iliopsoas tumor, malignant peripheral nerve sheath tumor (mpnst), neurofibromatosis type 1, nf1 mutation, pelvic mass, soft tissue sarcoma, sox10

## Abstract

Malignant peripheral nerve sheath tumors (MPNSTs) are malignant neoplasms of peripheral nerve sheath differentiation and may occur in association with neurofibromatosis type 1 (NF1). We report a case of a nine-year-old girl who presented with a rapidly enlarging left pelvic mass evolving over two months, accompanied by weight loss, pain, and functional limitation of the left lower limb with ipsilateral edema. Abdominopelvic imaging revealed a large heterogeneous mass centered on the left iliopsoas with pelvic and proximal thigh extension (108×100×151 mm), and thoracic staging identified bilateral pulmonary nodules suspicious for metastases. Careful dermatologic examination demonstrated numerous café au lait macules and axillary/inguinal freckling, strongly suggestive of NF1; targeted examination of the mother revealed similar pigmentary findings, consistent with previously unrecognized familial NF1. Biopsy showed a high-grade malignant spindle cell neoplasm with extensive necrosis and high mitotic activity. Immunohistochemistry was negative for desmin, myogenin, cytokeratin, and CD99, with focal SOX10 nuclear positivity, supporting the diagnosis of MPNST in the appropriate clinical and radiologic context. The patient was started on doxorubicin and ifosfamide chemotherapy. This case emphasizes that systematic cutaneous assessment in children with deep, rapidly growing soft tissue masses can be pivotal for identifying NF1, refining the differential diagnosis toward MPNST, and accelerating appropriate staging and multidisciplinary management.

## Introduction

Malignant peripheral nerve sheath tumors (MPNSTs) are rare, aggressive malignancies of peripheral nerve sheath differentiation and account for approximately 5-10% of soft tissue sarcomas [1]. A key clinical context for MPNST is neurofibromatosis type 1 (NF1), a tumor predisposition condition in which MPNST represents one of the most feared complications. Up to 25-50% of patients with MPNST have NF1, and the estimated lifetime risk of developing MPNST among individuals with NF1 is around 8-13% [2,3]. NF1 remains primarily a clinical diagnosis, and the revised international consensus criteria emphasize pigmentary features, such as multiple café au lait macules and axillary/inguinal freckling, particularly in children [4]. Clinically, this underscores the value of a careful dermatologic examination when evaluating any atypical, deep, or rapidly enlarging soft tissue mass, as recognition of NF1 can immediately sharpen diagnostic suspicion for nerve sheath malignancy and prompt targeted evaluation of relatives. In this report, we describe an aggressive pelvic MPNST in a nine-year-old girl in whom the tumor led to recognition of familial NF1 features (patient and mother), highlighting the importance of meticulous cutaneous assessment in pediatric oncology.

## Case presentation

We report a case of a nine-year-old girl, born to non-consanguineous parents, who presented to our center with a progressively enlarging left pelvic mass noticed by her family two months prior to admission, associated with unintentional weight loss of 2 kg and reduced appetite. There was no fever, no reported urinary or gastrointestinal symptoms, and no history of trauma.

**Figure 1:**
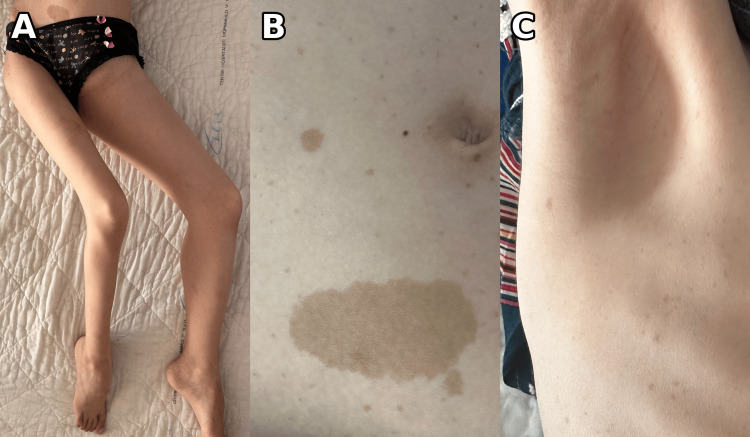
Clinical features suggestive of NF1 and posture related to the pelvic mass. (A) Antalgic posture with abduction of the left lower limb and flexion of the left knee in the context of a large left pelvic/iliopsoas mass. (B) Multiple café au lait macules on the trunk, including a large lesion. (C) Axillary freckling (lentigines). NF1: neurofibromatosis type 1

The patient was admitted, and on examination, she appeared slightly pale. Vital signs were as follows: temperature 37.3°C, heart rate 118 beats per minute (bpm) (mild tachycardia), respiratory rate 20/min, blood pressure 90/55 mmHg (mild hypotension), and oxygen saturation 98% on room air. Her weight was 27.5 kg and height 130 cm. On abdominal and pelvic examination, a solid, fixed, non-tender mass was palpated in the left iliac fossa, extending toward the anterosuperior aspect of the left thigh, with an estimated clinical size of 10 cm and no overlying inflammatory signs. There was a clear asymmetry between the two lower limbs, with enlargement of the left lower limb compared with the right, related to non-pitting edema. The left lower limb was maintained in an antalgic posture characterized by abduction of the limb and flexion of the left knee (Figure 1, panel A). Pain was elicited on manipulation of the left lower limb, particularly around the knee; the range of motion of the left hip and knee was limited by pain. Distal pulses were palpable, with no sensory or motor deficit on neurological examination. No peripheral lymphadenopathy was noted. Cardiovascular and pleuropulmonary examinations were unremarkable.

Dermatologic examination revealed multiple café au lait macules scattered across the body (approximately 20 lesions >5 mm, largest 63 mm) along with axillary and inguinal freckling (lentigines), suggesting neurofibromatosis type 1 (NF1) (Figure 1, panels B and C). No family history of NF1 was known at presentation; however, following the child’s evaluation, a targeted examination of the mother revealed multiple café au lait macules, although she had never been previously assessed or diagnosed with NF1. Pubertal assessment showed Tanner stage S2P1, and the patient had not yet reached menarche. Genetic testing for NF1 was not performed because molecular studies were not available in our setting.

Laboratory evaluation showed microcytic hypochromic anemia, leukocytosis with neutrophilia, mild thrombocytosis, and an inflammatory syndrome (elevated C-reactive protein at 74.18 mg/L {normal range: 0-5 mg/L} and erythrocyte sedimentation rate {ESR} at 60 mm/h), with elevated lactate dehydrogenase (LDH), while renal function, liver function tests, electrolytes, and serum albumin were within normal limits (Table 1).

**Table 1:** Laboratory findings on admission. Hb: hemoglobin; Hct: hematocrit; MCV: mean corpuscular volume; MCH: mean corpuscular hemoglobin; MCHC: mean corpuscular hemoglobin concentration; PLT: platelets; WBC: white blood cell count; ANC: absolute neutrophil count; GGT: gamma-glutamyl transferase; LDH: lactate dehydrogenase; ALP: alkaline phosphatase; AST: aspartate aminotransferase; ALT: alanine aminotransferase

Parameters	Result	Reference range
Hb	10.9 g/dL	12-16 g/dL
Hct	32.4%	37-47%
MCV	70.90 fL	80.00-98.00 fL
MCH	23.90 pg	27.00-32.00 pg
MCHC	33.60%	32.00-36.00%
PLT	426,000 cells/µL	150,000-400,000 cells/µL
WBC	12,820 cells/µL	4,000-10,000 cells/µL
ANC	9,100 cells/µL	1,500-7,000 cells/µL
Lymphocytes	2,760 cells/µL	1,000-4,000 cells/µL
Monocytes	740 cells/µL	200-800 cells/µL
Eosinophils	180 cells/µL	0-500 cells/µL
Basophils	40 cells/µL	0-200 cells/µL
CRP	74.18 mg/L	0-5 mg/L
Blood urea	0.14 g/L	0.10-0.30 g/L
Creatinine	5.56 mg/L	5.7-11.1 mg/L
Albumin	39.00 g/L	35-50 g/L
Uric acid	34.20 mg/L	26-60 mg/L
Calcium	88 mg/L	88-108 mg/L
Chloride	103 mEq/L	98-107 mEq/L
Phosphate	37.6 mg/L	23.0-47.0 mg/L
Total protein	68 g/L	64-83 g/L
Potassium	4.5 mEq/L	3.5-5.1 mEq/L
Sodium	139 mEq/L	138-145 mEq/L
Triglycerides	0.66 g/L	<1.50 g/L
GGT	16 U/L	9-36 U/L
LDH	349 U/L	125-243 U/L
ALP	101 U/L	<500 U/L
AST	15 U/L	5-34 U/L
ALT	7 U/L	0-55 U/L

**Figure 2:**
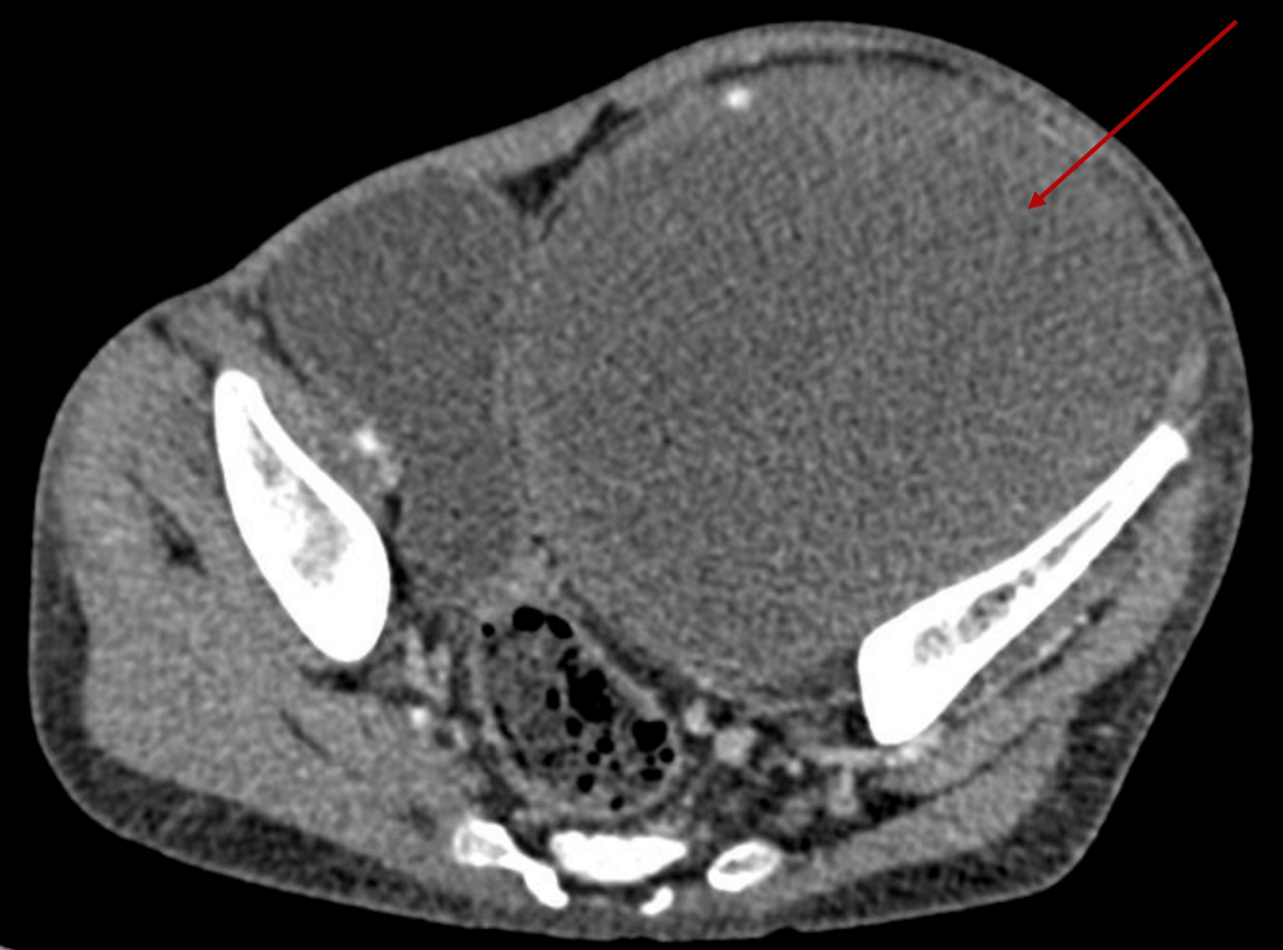
Axial abdominopelvic CT demonstrating a large pelvic soft tissue mass. Axial abdominopelvic CT image showing a large heterogeneous pelvic soft tissue mass (red arrow), occupying most of the pelvis and causing mass effect on adjacent structures.

Abdominopelvic ultrasound demonstrated a left-sided pelvic soft tissue mass with abdominal extension, in close proximity to the left coxal bone. Contrast-enhanced abdominopelvic CT scan revealed a large lesion centered on the left iliopsoas muscle, with pelvic, abdominal, inguinal, and anterosuperior extension to the left thigh. The mass measured 108×100×151 mm and had a mixed cystic solid component (Figure 2).

Given the patient’s age and the aggressive appearance of the pelvic mass, the initial differential diagnoses included rhabdomyosarcoma, Ewing sarcoma, and lymphoma. However, the presence of clinical features suggestive of NF1 raised suspicion for a tumor of peripheral nerve sheath origin.

**Figure 3:**
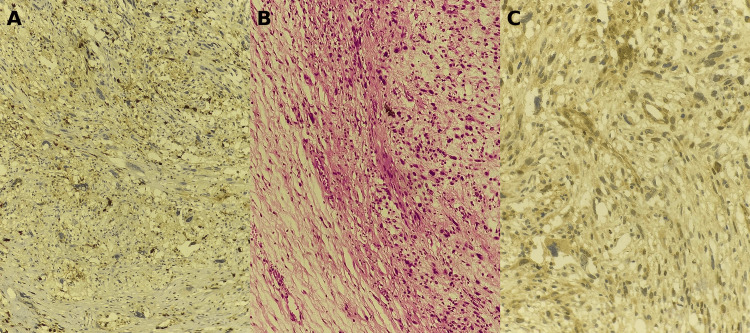
Histopathology and immunohistochemistry findings of the pelvic tumor. (A) Heterogeneous (patchy) positive staining with the anti-S100 antibody. (B) Hematoxylin and eosin (H&E) stain showing a spindle cell tumor proliferation with alternating hypocellular and hypercellular areas (×20). (C) Focal/patchy nuclear positivity of tumor cells with the anti-SOX10 antibody.

A biopsy of the pelvic mass was performed. Immunohistochemistry showed heterogeneous (patchy) S100 positivity (Figure 3A). Histology demonstrated a high-grade malignant spindle cell neoplasm with alternating hypocellular and hypercellular areas, extensive tumor necrosis, and an estimated mitotic activity of 10 mitoses per 10 high-power fields (Figure 3B). Additional immunohistochemistry showed negative staining for desmin, myogenin, cytokeratin, and CD99, while SOX10 demonstrated focal/patchy nuclear staining (Figure 3C). Taken together, and in correlation with the clinical and radiologic context, these findings supported the diagnosis of malignant peripheral nerve sheath tumor (MPNST).

**Figure 4:**
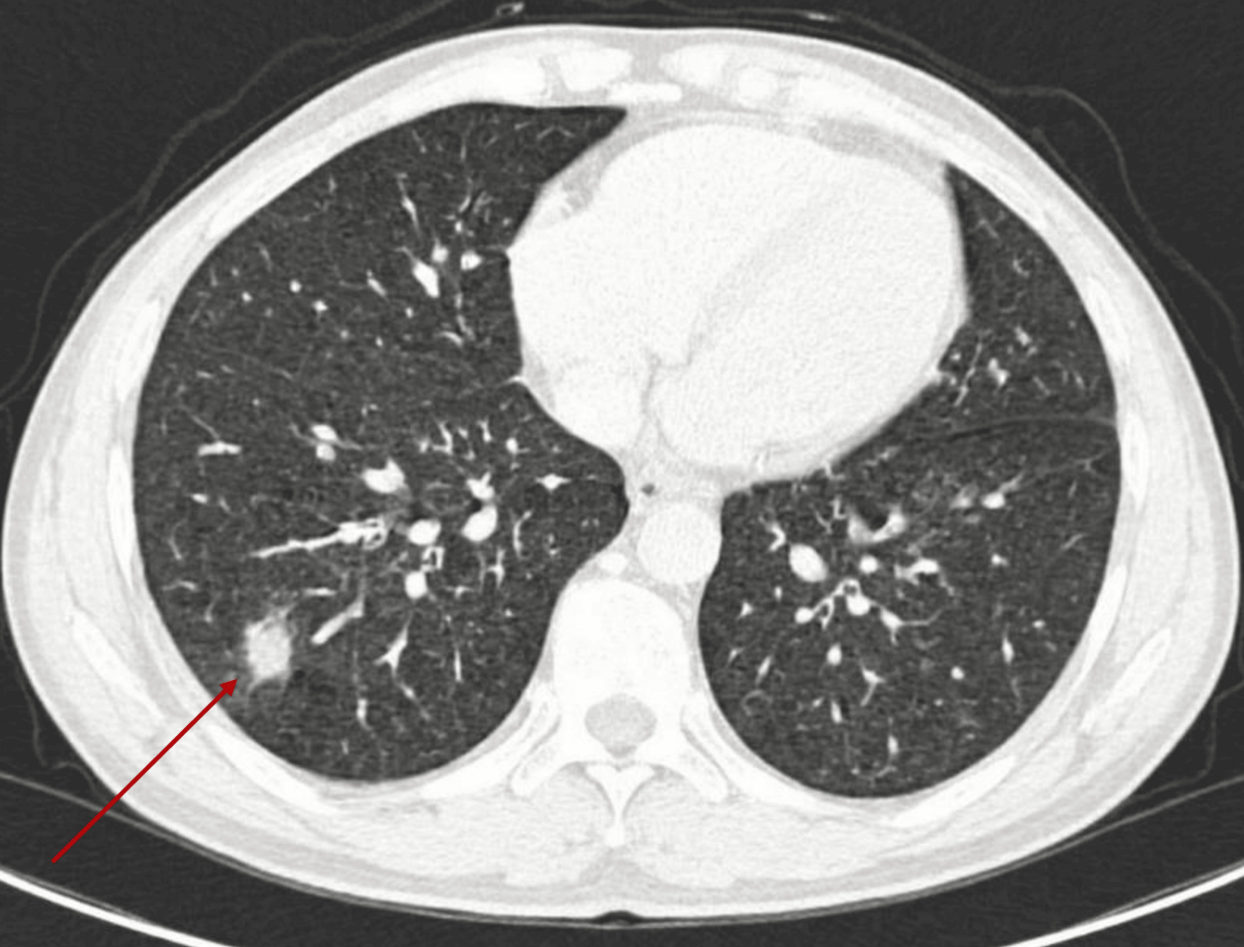
Chest CT demonstrating pulmonary nodule. Axial chest CT image obtained during initial staging showing pulmonary nodule in the lower lobes (red arrow), suspicious for metastatic involvement in the context of malignant peripheral nerve sheath tumor.

A dedicated cervical-thoracic CT scan performed for staging demonstrated bilateral lower-lobe pulmonary nodules, with the largest described as a right lower lobe ground-glass nodular infiltrate measuring 8 mm (Figure 4). Following multidisciplinary tumor board discussion (RCP), the pulmonary nodules were considered metastatic disease. As part of the metastatic workup, two bone marrow aspirates and two bone marrow biopsies were normal, with no evidence of marrow involvement. Ophthalmologic examination showed no Lisch nodules, and visual acuity was 10/10 bilaterally. The patient started doxorubicin and ifosfamide chemotherapy every 21 days as follows: doxorubicin 38 mg/day (38.1 mg/m²/day) IV on days one to two and ifosfamide 1.8 g/day (1.81 g/m²/day) IV on days one to five, with mesna uroprotection and adequate hydration.

## Discussion

MPNST is an uncommon and aggressive malignancy and represents only a small fraction of soft tissue sarcomas (approximately 5-10%) [1]. The overall annual incidence of MPNST is estimated at 1.46 cases per million people. In children, however, the rate is substantially lower, at about 0.56 per million among those younger than 15 years, with most pediatric cases occurring during adolescence, particularly between 10 and 19 years of age [5,6]. Our case is notable for the very young age (nine years). A substantial proportion of MPNSTs arise in the setting of neurofibromatosis type 1 (NF1), reported in the range of 25-50% in large series and reviews. For individuals with NF1, the estimated lifetime risk of developing MPNST is commonly reported around 8-13%, making it one of the most serious NF1-associated complications [2,3]. This elevated risk underlines why a rapidly enlarging, deep-seated mass in a patient with NF1 features should prompt urgent evaluation for malignant transformation.

Although molecular testing can provide confirmatory evidence by identifying a pathogenic NF1 variant (and is incorporated into the updated criteria), NF1 is still frequently diagnosed clinically using established criteria (including the 2021 international consensus revision) [4]. In children, pigmentary findings such as multiple café au lait macules and axillary/inguinal freckling carry strong diagnostic weight within the clinical criteria framework [4]. In our patient, the combination of numerous café au lait macules and axillary/inguinal freckling was highly suggestive of NF1; importantly, family history was not initially recognized, but a targeted post hoc examination of the mother revealed multiple café au lait macules, supporting a clinically suspected familial NF1 background even in the absence of a previous diagnosis. This point reinforces the practical message that a careful cutaneous examination (and a directed family assessment when NF1 is suspected) can meaningfully reframe the differential diagnosis of pediatric soft tissue masses and raise suspicion for nerve-sheath malignancy. NF1 is caused by inactivating variants in NF1, a tumor suppressor gene encoding neurofibromin, a negative regulator of RAS signaling [7]. In NF1, benign peripheral nerve sheath tumors, particularly plexiform neurofibromas, can serve as precursor lesions. Malignant transformation is understood as a multistep process initiated by biallelic NF1 inactivation in Schwann cell lineage cells, followed by additional genetic/epigenetic events (for example, alterations involving CDKN2A and PRC2 components), enabling progression along a continuum from benign plexiform neurofibroma to MPNST [7,8].

MPNST often presents a diagnostic delay, particularly when it arises in deep locations (pelvis/retroperitoneum), because early symptoms may be non-specific and the mass can enlarge before becoming clinically evident [9]. In patients with suspected or confirmed NF1, expert surveillance guidance highlights clinical “red flags” that should prompt urgent evaluation for malignant transformation, notably rapid growth/change in growth rate, new or persistent pain, and/or new neurologic deficits [10]. In our patient, the presentation was consistent with this pattern as follows: a progressively enlarging deep pelvic/iliopsoas mass associated with pain and antalgic posture, suggesting local mass effect and delayed recognition until functional impairment occurred. Imaging is central to evaluation, but morphology alone can be insufficient to reliably distinguish benign from malignant peripheral nerve sheath tumors [9]. In our case, CT demonstrated a large heterogeneous mass with a mixed cystic-solid component, which aligns with commonly reported malignant features such as heterogeneity and necrotic/cystic degeneration in MPNST [11,12]. Although CT was used here, MRI is generally preferred for local assessment and characterization of peripheral nerve sheath tumors, and a systematic review/meta-analysis supports MRI’s role in differentiating benign from malignant lesions (while acknowledging imperfect specificity) [11]. When malignant transformation is suspected in NF1, current guidance supports incorporating regional MRI plus fluorine-18 fluorodeoxyglucose (18F-FDG PET) (PET/CT or PET/MRI), as PET can help identify metabolically active targets for biopsy and improve diagnostic confidence [10,13].

In a pediatric malignant spindle cell tumor, the priority is to exclude common mimics. Rhabdomyosarcoma typically shows skeletal muscle differentiation with desmin and nuclear myogenin/MyoD1 positivity, whereas Ewing sarcoma commonly shows strong membranous CD99 expression and is ultimately confirmed by molecular testing when needed [14,15]. In our case, the tumor was negative for desmin, myogenin, cytokeratin, and CD99, making rhabdomyosarcoma, epithelial malignancy, and Ewing sarcoma unlikely. MPNST has no single pathognomonic marker; diagnosis relies on clinicopathologic correlation and exclusion of key differentials using an immunohistochemical panel. Focal nuclear SOX10 positivity supported Schwannian/nerve sheath differentiation in the appropriate clinical context (NF1 features), whereas focal synaptophysin was considered an ancillary, non-specific finding rather than lineage-defining [16]. MPNST generally carries an unfavorable prognosis, driven by its high risk of local recurrence and distant metastasis; outcomes are particularly poor once the disease is metastatic. In NF1-associated MPNST, survival is worse than in sporadic cases in pooled analyses, supporting closer vigilance and early intervention in NF1 patients [9,17].

From a management perspective, complete surgical resection with negative margins (R0) remains the only potentially curative treatment for localized disease [9,18]. However, pelvic/retroperitoneal locations often present late and can be difficult to resect upfront, which frequently necessitates multimodal decision-making. In unresectable, locally advanced, or metastatic MPNST, systemic therapy is typically adapted from soft tissue sarcoma standards, most commonly anthracycline-based chemotherapy, with ifosfamide often used in combination or sequentially depending on the clinical goal (tumor shrinkage for surgery vs. disease control) [18,19]. In our patient, the large deep pelvic tumor and pulmonary nodules at staging placed her in a high-risk setting.

## Conclusions

This case highlights an unusually early presentation of an aggressive pelvic malignant peripheral nerve sheath tumor (MPNST) in a nine-year-old girl, with pulmonary metastases at diagnosis. Importantly, meticulous skin examination revealing café au lait macules and axillary/inguinal freckling led to recognition of previously undiagnosed familial NF1 (in both the patient and her mother), promptly reframing the differential toward a nerve sheath malignancy. Our report underscores that in any child with a deep, rapidly enlarging mass, systematic assessment for NF1 cutaneous stigmata can be pivotal for timely diagnosis, staging, and management.
